# PKDL—A Silent Parasite Pool for Transmission of Leishmaniasis in Kala-azar Endemic Areas of Malda District, West Bengal, India

**DOI:** 10.1371/journal.pntd.0004138

**Published:** 2015-10-20

**Authors:** Swagata Ganguly, Pabitra Saha, Moytrey Chatterjee, Surajit Roy, Tamal Kanti Ghosh, Subhasish K. Guha, Pratip K. Kundu, Dilip K. Bera, Nandita Basu, Ardhendu K. Maji

**Affiliations:** 1 Department of Microbiology, Calcutta School of Tropical Medicine, Kolkata, West Bengal, India; 2 Department of Microbiology, NRS Medical College & Hospital, Kolkata, West Bengal, India; 3 Department of Zoology, A. P. C. Roy Govt. College, Himachal Bihar, Matigara, Siliguri, West Bengal, India; 4 Medinipur Medical College, West Medinipur, West Bengal, India; 5 Department of Tropical Medicine, Calcutta School of Tropical Medicine, Kolkata, West Bengal, India; 6 Department of Pathology, Calcutta School of Tropical Medicine, Kolkata, West Bengal, India; Hospital Universitário Professor Edgard Santos, BRAZIL

## Abstract

Post Kala-azar Dermal Leishmaniasis (PKDL) is a chronic but not life-threatening disease; patients generally do not demand treatment, deserve much more attention because PKDL is highly relevant in the context of Visceral Leishmaniasis (VL) elimination. There is no standard guideline for diagnosis and treatment for PKDL. A species-specific PCR on slit skin smear demonstrated a sensitivity of 93.8%, but it has not been applied for routine diagnostic purpose. The study was conducted to determine the actual disease burden in an endemic area of Malda district, West Bengal, comparison of the three diagnostic tools for PKDL case detection and pattern of lesion regression after treatment. The prevalence of PKDL was determined by active surveillance and confirmed by PCR based diagnosis. Patients were treated with either sodium stibogluconate (SSG) or oral miltefosine and followed up for two years to observe lesion regression period. Twenty six PKDL cases were detected with a prevalence rate of 27.5% among the antileishmanial antibody positive cases. Among three diagnostic methods used, PCR is highly sensitive (88.46%) for case confirmation. In majority of the cases skin lesions persisted after treatment completion which gradually disappeared during 6–12 months post treatment period. Reappearance of lesions noted in two cases after 1.5 years of miltefosine treatment. A significant number of PKDL patients would remain undiagnosed without active mass surveys. Such surveys are required in other endemic areas to attain the ultimate goal of eliminating Kala-azar. PCR-based method is helpful in confirming diagnosis of PKDL, referral laboratory at district or state level can achieve it. So a well-designed study with higher number of samples is essential to establish when/whether PKDL patients are free from parasite after treatment and to determine which PKDL patients need treatment for longer period.

## Author Summary

A significant number of PKDL patients have been detected in the endemic areas of Malda who would remain undiagnosed without active mass surveys. Such active survey is required in other endemic areas of the country to attain the ultimate goal of eliminating Kala-azar from this part of the world by reducing the source of infection. PCR-based method is helpful in confirming diagnosis of PKDL, which is not applicable at field level. A referral laboratory at district or state level can be a solution to the problem. Since PKDL cases do not have a fatal outcome, treatment administration of these cases can be deferred until confirmed results are obtained, which requires about 7 days’ time. The detection of leishmanial DNA in skin lesions during successive treatment courses is essential to establish whether/ when PKDL patients are parasite free during/after treatment and to determine which PKDL patients need treatment for a longer period.

## Introduction

The leishmaniasis is poverty related neglected tropical diseases [1] that have a major impact on health worldwide. Post kala-azar dermal leishmaniasis (PKDL), a sequel of leishmaniasis generally develops after apparent successful cure from visceral leishmaniasis (VL) [2, 3].

PKDL is a dermal manifestation characterised by macular, maculopapular or nodular rash caused by *L*. *donovani*. It occurs in about 10–20% of successfully treated visceral leishmaniasis (VL) cases in India [4, 5] approximately 50–60% of such patients in Sudan [6] and also in 2% of PKDL cases having no history of kala-azar [7]. Unlike VL, PKDL is not a life threatening condition. Its cosmetic values do not bother the poor patients who do not seek medical care and thus maintain the parasite in the population.

Three countries—Bangladesh, India, and Nepal signed a Memorandum of Understanding to eliminate VL from the Indian subcontinent by 2015 [8].

PKDL is one of the most important obstacles to achieve this goal. In South Asia, transmission of VL is anthroponotic, Post kala-azar dermal leishmaniasis is considered to be an important reservoir for VL [5, 6, 9], although little is known about the magnitude of the disease burden, its role in transmission and the risk it may pose to the elimination programme [10].

Observations suggest that the duration, dose and type of drug may affect the occurrence of PKDL, e.g. the incidence of PKDL is higher after treatment with sodium stibogluconate [5–7, 11, 12], compared to amphotericin B, Miltefosine [13] and paramomycin [14–17].

Diagnosis and treatment of PKDL cases are challenges faced by the programme [18]. Confirmation of diagnosis is difficult and. microscopic demonstration of Leishman-Donovan bodies from skin lesions has low sensitivity [6, 19–21]. The PCR-based molecular diagnostic method is anticipated to provide a powerful approach to overcome the problem [11, 22–26].The use of PCR as a diagnostic tool has not achieved wide spread application in field level.

The treatment of PKDL is complex and not standardized requiring long courses of therapy [18]. There is paucity of prognostic marker other than clinical disappearance of skin lesions. More effective, shorter-duration treatment regimens and PKDL diagnostic testing adaptable to community-based active case finding are urgently needed to enable the regional VL elimination initiative to succeed. The present study has been designed to determine the actual disease burden in a VL endemic area of Malda district, West Bengal and comparison of the three diagnostic tools–LD body demonstration (smear, culture), RDT (rK-39) and PCR-based molecular method for PKDL case detection and to observe the regression pattern of lesions following treatment.

## Materials and Methods

### Study area and population

The study was conducted in seven tribal villages of three sub-centres (1 from Habibpur and 2 from Gazole block) of Malda. The total sub-centres of these two study blocks are 103 among them 81 sub-centres are endemic for leishmaniasis.

### Mass survey

In the beginning sensitization of the community was conducted involving the local health staff, Panchayat members and also the local health authorities. The objectives and activities of the project were explained to the study population. Active mass screening was done by house-to-house survey for collecting demographic data of all individuals. The study teams were asked to fill up a questionnaire regarding history of kala-azar and treatment received along with other demographic parameters. All available individuals were screened by RDT (based on detection of antileishmanial antibody) for the detection of anti-leishmanial antibody after obtaining verbal consent. All rk39 positive cases with or without history of kala-azar but having signs of PKDL and hence considered as probable PKDL cases were examined clinically.

Slit skin scrapings were collected from these probable patients after obtaining written informed consent for diagnosis by three different methods on day0 and on completion of treatment.

### Laboratory methods

#### Collection of slit skin scraping for smear, culture, and PCR

The affected area of the skin was cleaned with 70% v/v alcohol and allowed to dry completely. The margin of the lesion was squeezed firmly between the finger and the thumb to drain the area of blood. Using a sterile scalpel blade, a small incision was made into the dermis. The cut surface was then scraped in an outward direction to obtain the tissue fluid and cells for smear, culture in NNN media and in NET buffer (150 Mm Nacl, 15 Mm Tris-HCl [pH-8.30], 1mM EDTA) for DNA isolation for PCR.

#### Microscopy and culture

One part of the skin scraping sample was aseptically inoculated into NNN media and incubated at 22°C for 3 weeks. A drop of the media fluid was examined under microscope for demonstration of promastigotes. Smear was prepared on clean glass slide from another part of the scraping sample and was examined by two experts following Giemsa staining.

#### Examination for *M*. *leprae*

Two experts examined one Ziehl-Neelsen stained slit skin smears from each patient to exclude *Mycobacterium leprae*.

#### DNA isolation

DNA was isolated from skin scrapings collected in NET buffer using Qiagen DNA mini kit as per manufacturer’s instructions.

#### PCR as diagnostics

For diagnosis of parasite in clinical samples *L*. *donovani* kinetoplast DNA was amplified by primary and nested PCR strategies as described earlier [23, 24] with minor modifications. All amplification reactions were carried out in a final volume of 50 μl. The oligonucleotide primers and PCR conditions used in both the primary and nested reaction are summarized in Table 1. All necessary precautions were taken to avoid contamination. During each round of PCR amplification, DNA of a laboratory maintained field isolate in NNN media was used as positive control.

**Table 1:** Primers and profiles used for PCR amplification of the *L*. *donovani* kinetoplast DNA.

			PCR programme						
			Denaturation		Annealing		Elongation		
PCR reaction	Primer sequence (5ʹ–3ʹ)	Mg^+2^ conc. (mM)	Temp. (^0^C)	Time (min)	Temp. (^0^C)	Time (min)	Temp. (^0^C)	Time (min)	No. of cycle
**Primary**	AAATCGGCTCCGAGGCGGGAAAC	1.5	94	1	45	1	72	2	40
	GGTACACTCTAT CAGTAGCAC								
**Nested**	TCGGACGTGTGTGGATATGGC	1.5	94	1	49	1	72	1.5	35
	CCGATAATATAGTATCTCCCG								

### Treatment and follow-up

As per National Vector Born Disease Control Program (NVBDCP) guidelines, rK 39 positive individuals with signs and symptoms of PKDL were treated irrespective of their parasitological and molecular biological results. All such patients were referred to their respective Block Primary Health Centre (BPHC) for treatment. During first part of the study miltefosine was unavailable so 10 patients were treated with SSG and remaining 16 cases were with miltefosine. Drugs were supplied by Dy. Director of Health Services, Malaria, State Programme Officer, Government of West Bengal. The dosage of the above mentioned drug administered was according to WHO guidelines as follows: SSG was given at a dose of 20 mg/kg IM for 90–120 days and Miltefosine orally for 12 weeks at dose—children aged 2–11 years, 2.5 mg/kg per day, 12 years and < 25 kg body weight, 50 mg/day, 25–50 kg body weight, 100 mg/day, > 50 kg body weight, 150 mg/day.

Though the treatment was done by the respective BPHC but the treatment was supervised by trained local volunteers and/or study team members. All enrolled patients were examined clinically and biochemically after each treatment course and examined clinically once in a month for 1.5 years post-treatment period.

### Case definitions

Probable PKDL is a patient from an area endemic for kala-azar with multiple hypopigmented macules, papules or plaques or nodules with no sensitivity loss. Confirmed PKDL is a patient from an area endemic for kala-azar with multiple hypopigmented macules, papules, plaques or nodules who is positive for rk39 test and/ or parasite- or PCR-positive in a slit skin smear. A Cure case is defined as complete disappearance of skin lesion(s) after treatment, as reported by the patient and assessed by the clinician and also negative by PCR.

### Data analysis

Z test was used to compare the sensitivity of 3 diagnostic methods. GraphPad InStat3 (version 3.06) statistical software was used for determination of Median, Range, 95% CI. Biochemical parameters values (before, during and after treatment) were by compared by ANOVA.

### Ethical consideration

Before initiation of data collection, a team headed by a doctor from Calcutta School of Tropical Medicine explained the objectives of the study and the benefit that they would receive from the study to each head of the family and other members. They were also informed that their identity would not be disclosed and they could withdraw from the study at any time without any explanation. After such explanation they were invited to participate into the study. When they agreed, team leader noted it and then the team members recorded the demographic data like age, sex, body weight; past history of VL/PKDL, treatment regimen, mode of treatment and duration of treatment in a data collection note book. It was hardly possible to obtain written informed consent from all individuals who consented to participate in the study. Each individual was screened for antileishmanial antibody from a drop of finger prick blood at the field site with appropriate precautions. Written informed consent was obtained from each suspected PKDL cases or from their parents/legal guardian for all child patients for collection of slit skin samples for smear/culture, PCR and also for collection of 5 ml venous blood for biochemical studies on day 0 and after each course of treatment. The Ethics Committee of the Calcutta School of Tropical Medicine has approved the ethical considerations undertaken at the field site in the study.

## Results

### Prevalence of PKDL in study area

**Fig 1:**
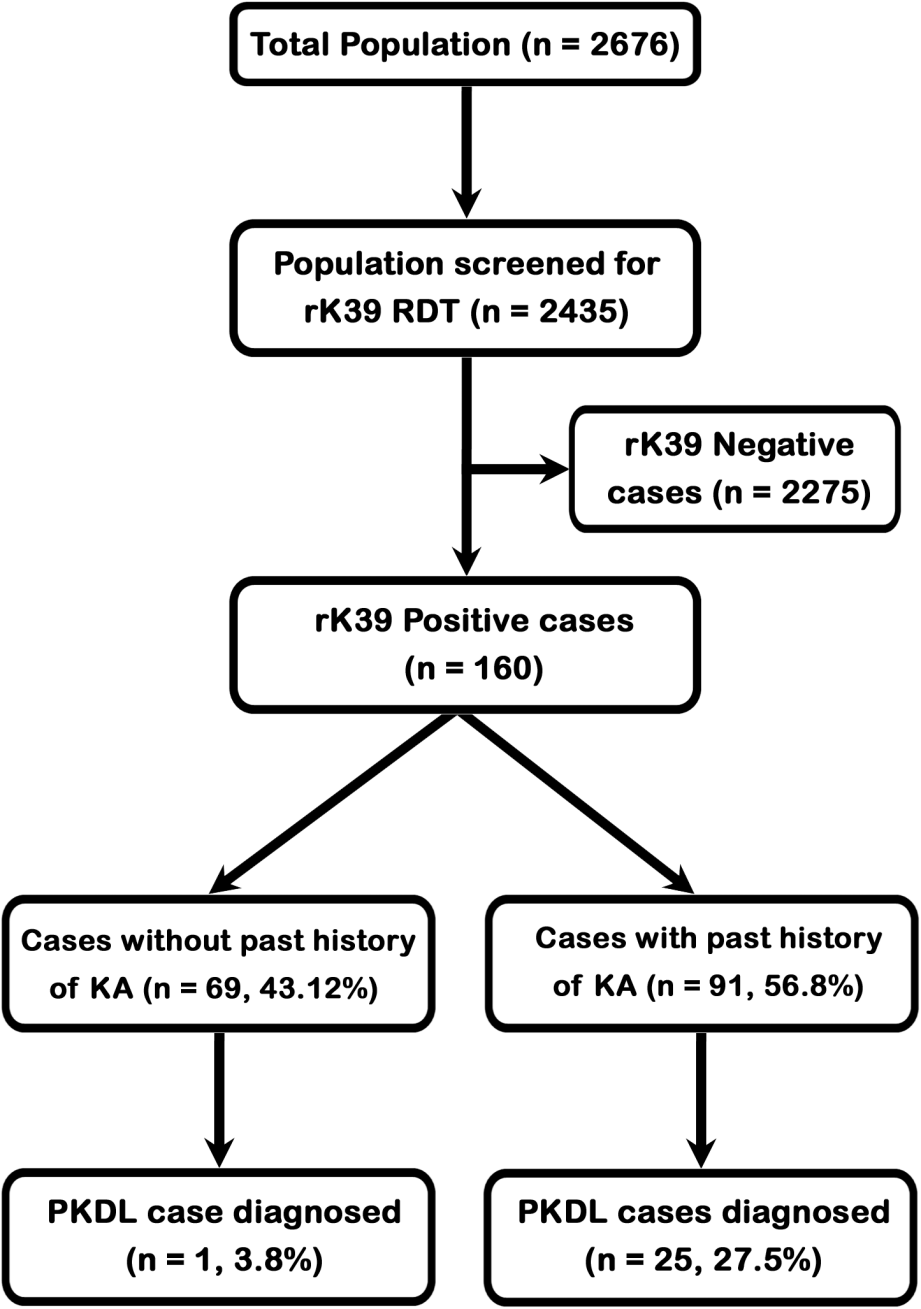
Showing the results of population screening.

Active mass survey was done in seven villages of Gazole and Habibpur blocks of Malda district during August- October, 2012. Total population of the study villages was 2676 among them 2435 population was screened for antileishmanial antibody. A total of 160 individuals were positive for antileishmanial antibody of them 91 had a history of Kala-azar and 69 without any history of Kala-azar. Among 91 individuals having past KA history 25 (27.5%) had signs of PKDL. Interestingly one patient without any past history was positive for antileishmanial antibody and had macular type of lesions all over body which was confirmed as PKDL by demonstrating parasitic DNA by PCR (Fig 1). There were another 15 cases with similar type of skin lesions as that of PKDL but were negative for antileishmanial antibody by RDT and leishmanial DNA by PCR, were referred to local Block Primary Health Centres for their management. Among them Pityriasis alba in four cases, Pityriasis versicolor in eight cases and Seborrhoeic dermatitis in three cases were diagnosed and treated accordingly. So an overall prevalence of PKDL was 16.25% (26/160) among the people who were positive for antileishmanial antibody. Among 26 PKDL cases, 25 had past history of VL and one without such past history. Slit skin smears from all 41 (26 PKDL and 15 non-PKDL cases) patients with skin lesions were found to be negative for acid fast bacilli.

All 26 patients were positive for antileishmanial antibody by rK-39 rapid diagnostic kit. Parasite was demonstrated in 4 cases only with a sensitivity rate of 15.38% of whom 3 were with nodular lesions and one was papular. Parasitic DNA was detected by PCR in 23 cases with a sensitivity rate of 88.46%. No significant difference was noted between PCR and RDT (Z = 0.3936, P = 0.6939). Microscopy was found to be less sensitive than both RDT (Z = 3.3201, P = 0.0009) and PCR (Z = 2.9478, P = 0.0032).

### Characteristics of PKDL patients

All 26 patients with or without having past history of VL were examined clinically. On examination sign of VL like hepato-splenomegaly, fever or history of fever during last one month were not recorded in any case. Anaemia was not detected as evidenced by absence of pallor. So, all of them were diagnosed as PKDL. Among 26 PKDL cases 14 were male and 12 were female with an age ranging from 5 years to 42 years. History of kala-azar was more than 5 years in 10 patients and 15 had within 5 years. The average interval between kala-azar treatment and development of PKDL lesions was 3.13 years ranging from 6 months to 14 years. Macular and papular lesions were observed in 23 cases whereas polymorphic lesions (maculopapular and nodular) was found in three cases. The demographic parameters are given in Table 2.

**Table 2:** Demographic parameters of PKDL patients.

Characteristics	(n = 26)
**Sex: no. (%)**	
Male	14 (53.8)
Female	12 (46.2)
**Age category: no. (%)**	
< 15 yrs	8 (30.8)
> 15 yrs	18 (69.2)
**H/O past VL: no. (%) (n = 25)** #	
1–3 yrs	11 (44.0)
4–10 yrs	9 (36.0)
> 10 yrs	5 (20.0)
**Time to develop PKDL after VL treatment: no. (%) (n = 25)** #	
< 1 yr	3 (12.0)
1 - < 5 yrs	16 (64.0)
≥ 5 yrs	6 (24.0)
**Lesion types: no (%)**	
Hypopigmented patch	23 (88.5)
Hypopigmented patch + nodule	3 (11.5)
**Lesion distribution: No (%)**	
Face	2 (7.7)
Face, arms	2 (7.7)
Face, arms, legs	12 (46.2)
All over body	10 (38.5)

# There was one PKDL patient without previous history of VL.

### Treatment outcome and lesion regression pattern

First 10 patients were treated with SSG and other 16 with oral miltefosine. They were examined monthly during treatment and after 6 months 1 year and 1.5 years post-treatment period. One patient did not complete full course of treatment as he left the study area after 1^st^ month of miltefosine treatment. Among 3 cases with mixed lesions, nodular lesions resolved at the end of 3^rd^ month of treatment in 2 cases but in other case nodular lesion persisted up to six month post treatment period. In all 3 cases macular lesions dissolved slowly during 6 to 12 month post treatment period.

**Fig 2:**
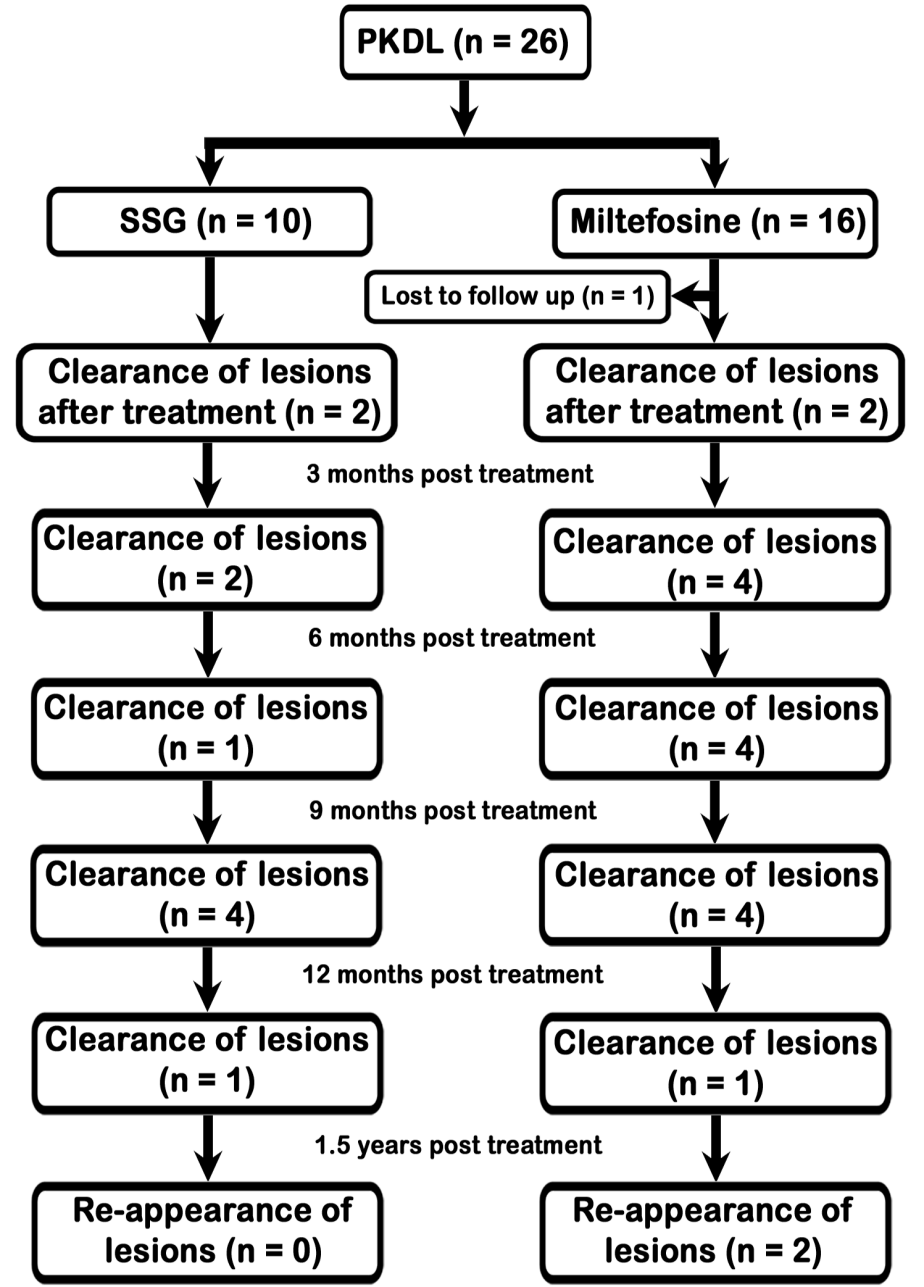
Lesion regression pattern of 25 treated PKDL cases.

Macular lesions gradually regressed and disappeared in 4 cases after treatment and in 11 after 6 months post-treatment. In seven cases macular lesions even persisted after 6 months post treatment but resolved within 1 year (Fig 2). Among the patients treated with miltefosine nausea was recorded among all of them, vomiting in 3 cases only. They were managed symptomatically with antiemetic drugs. Diarrhoea was noted in 2 patients and was treated with Oral Rehydration Solution (ORS), loperamide. The biochemical parameters like serum urea, creatinine, billirubin, SGPT, SGOT, and alkaline phosphatase levels before, during and at the end of therapy are shown in Table 3. There was no significant change in the levels of these laboratory tests before, during and after therapy as all P values were > 0.05. In 2 cases hepatic enzyme levels was increased by 2 folds, their doses of miltefosine were reduced from 150 mg/day to 100 mg/day as per WHO guidelines for 2 weeks; full dose was again administered after 2 weeks on documentation of normal enzyme levels. Patients who were treated with SSG complained of pain at injection site, myalgia, arthralgia were recorded in all 10 cases who were administered non-steroidal anti-inflammatory drugs. Injection was given on 2 arms and 2 gluteal muscles alternately to minimise pain at injection site.

**Table 3:** Biochemical parameters of PKDL patients treated with miltefosine and SSG.

	Values*								
		Miltefosin arm (n = 16; Male = 9, Female = 7; ≤ 15 yrs = 5, > 15 yrs = 11)				SSG arm (n = 10; Male = 5, Female = 5; ≤ 15 yrs = 3, > 15 yrs = 7)			
Biochemical Parameters	Normal	Before treatment	After 1 month of treatment	After 2 months of treatment	Completion of treatment	Before treatment	After 1 month of treatment	After 2 months of treatment	Completion of treatment
**Serum Urea (mg/dl)**	**10.0–50.0**								
Median		18.00	18.00	19.00	18.00	25.00	25.00	24.00	24.00
Range		11.00–31.00	12.00–24.00	12.00–24.00	12.00–25.00	16.00–33.00	19.00–28.00	18.00–33.00	19.00–36.00
95% CI		15.86–22.27	15.99–19.13	16.91–20.84	16.64–20.23	21.69–28.70	23.30–27.29	21.16–27.64	20.70–28.89
**Serum Creatinine (mg/dl)**	**0.39–1.20**								
Median		0.50	0.64	0.70	0.70	0.62	0.63	0.66	0.64
Range		0.50–0.90	0.50–1.00	0.50–1.20	0.50–1.00	0.42–0.90	0.44–1.00	0.50–1.20	0.48–0.98
95% CI		0.52–0.65	0.58–0.75	0.59–0.82	0.61–0.75	0.53–0.73	0.55–0.80	0.55–0.87	0.56–0.79
**Billirubin (mg/dl)**	**0–1.1**								
Median		0.44	0.34	0.35	0.35	0.46	0.42	0.45	0.44
Range		0.26–1.20	0.25–1.22	0.25–0.82	0.20–0.85	0.29–0.70	0.25–1.01	0.28–0.90	0.30–0.80
95% CI		0.39–0.69	0.32–0.63	0.31–0.50	0.33–0.52	0.38–0.61	0.35–0.70	0.35–0.65	0.38–0.62
**SGPT (U/L)**									
**Male**	**< 41**								
Median		35.00	31.00	34.00	38.00	37.00	32.00	42.00	37.00
Range		14.00–64.00	19.00–101.00	15.00–62.00	17.00–76.00	22.00–43.00	26.00–51.00	19.00–48.00	21.00–44.00
95% CI		19.42–47.03	20.88–65.56	25.28–47.83	23.59–50.63	23.12–44.08	22.74–46.46	24.75–54.05	24.07–45.93
**Female**	**< 31**								
Median		25.00	24.00	24.00	23.00	25.00	24.00	27.00	27.00
Range		12.00–93.00	17.00–62.00	11.00–66.00	13.00–49.00	18.00–29.00	20.00–38.00	22.00–47.00	23.00–40.00
									
95% CI		7.55–57.88	13.21–41.94	11.23–51.63	16.34–38.21	17.95–28.85	17.81–36.59	18.69–46.91	20.32–37.28
**SGOT (U/L)**									
**Male**	**< 35**								
Median		37.00	43.00	33.00	37.00	33.00	34.00	35.00	35.00
Range		26.00–54.00	34.00–73.00	27.00–66.00	31.00–58.00	29.00–35.00	31.00–42.00	24.00–38.00	29.00–45.00
95% CI		31.85–44.82	36.04–57.97	28.65–47.56	32.77–45.67	28.43–35.97	29.01–40.19	25.59–39.60	28.63–43.77
**Female**	**< 31**								
Median		28.00	29.00	26.00	26.00	26.00	28.00	27.00	27.00
Range		21.00–78.00	23.00–57.00	16.00–72.00	15.00–60.00	18.00–39.00	22.00–39.00	26.00–42.00	21.00–38.00
95% CI		17.29–53.00	21.41–42.59	13.56–49.29	17.39–43.75	18.49–37.91	21.48–36.92	21.63–38.37	20.29–35.70
**Alkaline Phosphatase (U/L)**									
**≤ 15 yrs**	**53–369**								
Median		279.00	265.00	275.00	262.00	287.00	297.00	310.00	278.00
Range		124.00–423.00	127.00–355.00	122.00–305.00	134.00–280.00	241.00–305.00	255.00–301.00	275.00–350.00	248.00–302.00
95% CI		145.02–444.58	145.24–363.16	154.42–335.18	164.94–311.86	195.67–359.66	221.03–347.64	218.44–404.90	208.78–343.22
**> 15 Yrs**	**42–128**								
Median		110.00	89.00	93.00	88.00	77.00	82.00	87.00	85.00
Range		38.00–164.00	56.00–130.00	54.00–128.00	59.00–133.00	48.00–120.00	55.00–109.00	52.00–115.00	60.00–100.00
95% CI		80.34–128.75	76.75–109.25	75.75–104.43	77.56–106.99	61.01–106.99	64.10–100.75	67.69–104.02	69.45–98.56

*There was no significant change in the levels of these laboratory tests before, during and after therapy as all P-values were > 0.05.

## Discussion

There are few reports of community-based study regarding prevalence of PKDL in endemic areas of India [7, 27]. In our study 25 patients developed PKDL out of 91 individuals having past history of VL with a prevalence of 27.47% which is higher than that reported from India [7, 27], Bangladesh [10] and Nepal [12]. These patients have all been detected by active survey during house-to-house visit. Among 26 PKDL cases, 3 patients had nodular forms from whom parasite could be detected quite easily, which implies that these lesions are more accessible to KA vector–sandfly. The role of nodular PKDL forms in transmission of kala-azar is significant [9].

Any delay in detection of macular forms of PKDL may have double impact: further transmission of PKDL by vector and subsequent development into nodular forms where parasites are easily accessible to the sandfly.

To achieve the goal of kala-azar elimination, early diagnosis by active mass survey and treatment is an important aspect. Proper diagnosis of PKDL cases in the field level which is primarily dependant on clinical manifestations is a real problem.

Previous reports indicated that microscopy had a detection rate between 4% to 58% for LD bodies in skin smears [6, 19, 20]. The sensitivity of this technique is low due to the very low number of parasites in slit skin smears and skin biopsy specimens. The rate of detection of PKDL cases by microscopy from skin scraping sample depends upon the selection of lesion site and collection of scraping sample which varies from person to person as the distribution of parasite throughout the lesion is not homogeneous. The low sensitivity of the diagnostic technique prolongs the time to diagnosis.

The immunochromatography test (ICT) with rK39, a recombinant antigen, has been found to be highly sensitive and specific for detection of antibodies in patients with VL and PKDL [28]. In the present study all suspected PKDL cases and those with past history of VL tested positive for anti-leishmanial antibody. Though this test is sensitive, diagnosis based on rK39 strip test is not conclusive for PKDL because of the persistence of anti-leishmanial antibodies after *L donovani* infections [29].

A PCR based diagnostic method for leishmaniasis, in which conserved sequences in leishmanial kinetoplast mini-circle DNA is amplified, has been developed in recent years. The PCR-based molecular diagnostic method is anticipated to provide a powerful approach to the diagnosis of leishmaniasis [22, 30, 31]. In the present study we detected that sensitivity of primary PCR was 65.4% (17 out of 26) which increased to 88.5% (23 out of 26) in nested PCR. Similar observation was also reported from different kala-azar endemic countries [19, 22–24]. PCR was negative in 15 cases with similar type of lesions which was difficult to differentiate clinically from PKDL lesions. Those cases were negative for antileishmanial antibody by rK-39 strip test. So in cases having no past VL history, rK-39 test is sufficient for differential diagnosis but in cases with history of VL, PCR is the only way to solve the problem. A referral laboratory at District or State level can be a solution to the problem. Since PKDL cases usually do not have a fatal outcome, hence treatment administration of these cases can be deferred till confirmed results are obtained which requires about 7 days’ time. Most of the macular lesions regressed slowly but persisted even after treatment completion which gradually disappeared during 6 to 12 months post-treatment period without further treatment.

In the present study, initial cure rate followed by SSG and mitefosine was 100% at one year post treatment follow up but at 1.5 years follow up lesion reappeared in two cases who were treated with miltefosine. In both the patients nodular lesions cleared rapidly but macular lesions regressed very slowly. Both of them were adults with age of 25 and 27 years respectively. No correlation was established with dose of the drug since they were treated with same dose as that of other adult patients. Earlier studies also reported miltefosine failure PKDL cases from India [32, 33]. So the patients treated with miltefosine need a long term follow up at least for 2–3 years. SSG has long been used as first line agent to treat leishmaniasis throughout all endemic countries. During early 2000s decreasing efficacy of the drug was reported from Indian subcontinent [34,35] in VL. SSG was also used in PKDL with a longer duration than VL. This long duration treatment with high volume of drug had practical problems with toxicity and either nonacceptance or poor compliance. Unresponsiveness has also been recorded against it [36]. Amphotericin B, miltefosine have been used either alone or in combination to treat leishmaniasis. Very recently NVBDCP introduced liposomal amphotericin B to treat both VL and PKDL. During first part of our study, Amphotericin B and miltefosine were not available for which first ten PKDL cases were treated with SSG and responded to treatment.

A definition of cure is important; an ideal would be a simple parasitological endpoint that could occur well before re-pigmentation and resolution of lesions, which may take much longer time. In the present study we observed that lesions persisted even after six months post treatment period in majority of patients. A well designed study based on PCR is the need of the hour to determine the time required to achieve parasite-free skin lesions in PKDL cases.
